# Robot-Assisted Electrode Insertion in Cochlear Implantation Controlled by Intraoperative Electrocochleography—A Pilot Study

**DOI:** 10.3390/jcm11237045

**Published:** 2022-11-29

**Authors:** Wojciech Gawęcki, Andrzej Balcerowiak, Paulina Podlawska, Patrycja Borowska, Renata Gibasiewicz, Witold Szyfter, Małgorzata Wierzbicka

**Affiliations:** 1 Department of Otolaryngology and Laryngological Oncology, Poznan University of Medical Sciences, 60-355 Poznan, Poland; 2 Department of Otolaryngology and Laryngological Oncology, Heliodor Swiecicki Clinical Hospital, 60-355 Poznan, Poland; 3 Institute of Human Genetics, Polish Academy of Sciences, 60-479 Poznan, Poland

**Keywords:** cochlear implant, robot, residual hearing, electrocochleography

## Abstract

Robotics in otology has been developing in many directions for more than two decades. Current clinical trials focus on more accurate stapes surgery, minimally invasive access to the cochlea and less traumatic insertion of cochlear implant (CI) electrode arrays. In this study we evaluated the use of the RobOtol^®^ (Collin, Bagneux, France) otologic robot to insert CI electrodes into the inner ear with intraoperative ECochG analysis. This prospective, pilot study included two adult patients implanted with Advanced Bionics (Westinghouse PI, CA, USA) cochlear implant, with HiFocus™ Mid-Scala electrode array. The standard surgical approach was used. For both subjects, who had residual hearing in the implanted ear, intraoperative and postoperative ECochG was performed with the AIM^TM^ system. The surgeries were uneventful. A credible ECochG response was obtained after complete electrode insertion in both cases. Preoperative BC thresholds compared to intraoperative estimated ECochG thresholds and 2-day postoperative BC thresholds had similar values at frequencies where all thresholds were measurable. The results of the ECochG performed one month after the surgery showed that in both patients the hearing residues were preserved for the selected frequencies. The RobOtol^®^ surgical robot allows for the correct, safe and gentle insertion of the cochlear implant electrode inside the cochlea. The use of electrocochleography measurements during robotic cochlear implantation offers an additional opportunity to evaluate and modify the electrode array insertion on an ongoing basis, which may contribute to the preservation of residual hearing.

## 1. Introduction

Robotics in otology has overtaken other fields of head and neck surgery and has been developing in many directions for more than two decades. Robots for otology can be classified as collaborative when intervention is constrained by the robot but the surgeon directly actuates the end-effector, teleoperated when a remotely controlled robot enables the tremor reduction, or autonomous when the surgeon monitors the robot performing a task [1,2,3]. Current clinical trials focus on more accurate stapes surgery, minimally invasive access to the cochlea and less traumatic insertion of cochlear implant (CI) electrode arrays. A robot-based holder may combine the benefits of endoscopic exposure with a two-handed technique. Robot-assisted endoscopy is a safe and trustworthy tool for several categories of middle ear procedures, such as myringoplasty, partial ossiculoplasty and total ossiculoplasty [4,5]. Robot-assisted manipulation of the ossicular chain in cadaveric temporal bones using a robotic arm (RobOtol^®^) was described as reliable [6]. Otosclerosis surgery with robotic assistance enhances the precise amplitude of motion and the surgeon’s dexterity and rapidly reduces the learning curve [7]. Moreover, the surgical simulator has been developed to plan new procedures that exploit the robot`s capacities, enhancing gesture accuracy and allowing exploration of new procedures for middle ear surgery [8].

Robot-assisted cochlear implantation is the result of over a decade of research & development work but is still in its childhood era [9,10]. Successful hearing rehabilitation with a CI is a complex, multi-stage process. “Clinical Practice Guidelines” are widely accepted for the standardization of such processes; however, there is still room for refining the diagnostic and technical steps for optimal results, which is where robotic surgery comes in [11]. As the first device to obtain European certification for clinical use (CE mark), the RobOtol^®^ system has been used in France and China since 2019 for robotic-assisted CI in profoundly deaf adults and children [5,6,12]. The beginning of research dates back to 2005, the commercial launch of RobOtol^®^ on the market in 2018 and soon after, in 2019, the first robotic cochlear implantation at the APHP Pitié-Salpêtrière Department took place. Recently the robotic system has been implemented in clinical practice [6,12,13,14] and the assumption was optimization of the electrode array insertion into the scala tympani (ST). The subject of discussion and the key question is how to compare and how to measure the superiority of a robotic electrode insertion over a manual one. This was performed based on the analysis of retrospective (manual insertion) and prospective (robotic) pair-matched patients based on imaging studies and on the results of speech rehabilitation [12,13,14].

One method of accurately assessment of electrode array placement in the cochlea is intracochlear electrocochleography (ECochG) [15,16]. Intracochlear ECochG is also a promising method for pre-curved electrodes [17]. In general, ECochG is a measurement technique based on recording electrical potentials generated by the inner ear and auditory nerve in response to acoustically evoked stimulation [18]. Contrary to the well-known and described standard extracochlear ECochG measurement techniques that require the use of surface electrodes, trans-tympanic or extra-tympanic electrodes, in intracochlear ECochG measurement application, the CI electrode array is used as the measuring electrode [17]. The ECochG response to low-frequency tone burst stimulus is mainly composed of the cochlear microphonic (CM) and the auditory nerve neurophonic (ANN) [19]. The CM is derived from the stereocilia of the outer hearing cells and follows the stimulus waveform [20]. The ANN is the electric potential correlate of phase-locking in the auditory nerve [19]. Therefore, monitoring of extracted CM electrical potentials allows indirect insight into the inner ear’s micromechanical activity and provides data for assessing electrode insertion trauma during the electrode array insertion and after cochlear implantation at subsequent follow-ups. Previous work has also demonstrated that ECochG recordings correlate with postoperative pure-tone thresholds in subjects with sensorineural hearing loss [21]. Additionally, CI recipients who show preserved residual hearing perform better than those without postoperative hearing [22].

Thus, we evaluated the use of the RobOtol^®^ otologic robot to insert CI electrodes into the inner ear with intraoperative ECochG (iECochG) analysis. The objective of the study was to clarify how the iECochG can improve the robotic cochlear electrode array insertion.

## 2. Materials and Methods

### 2.1. Study Design and Patients

**Figure 1:**
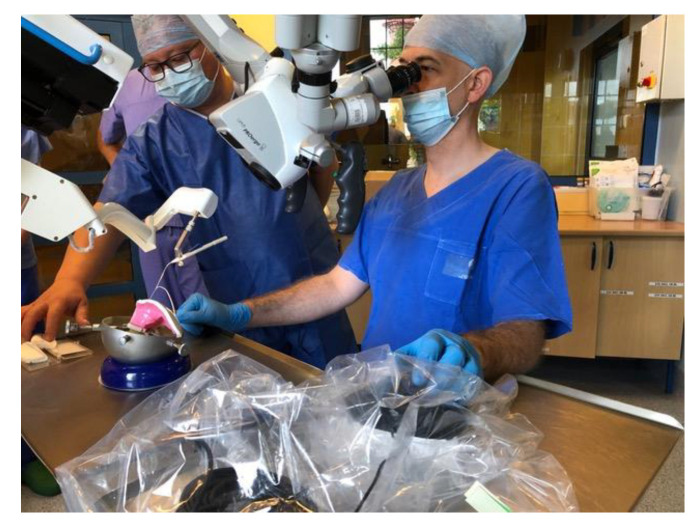
Surgical training with RobOtol^®^ on artificial temporal bone.

**Figure 2:**
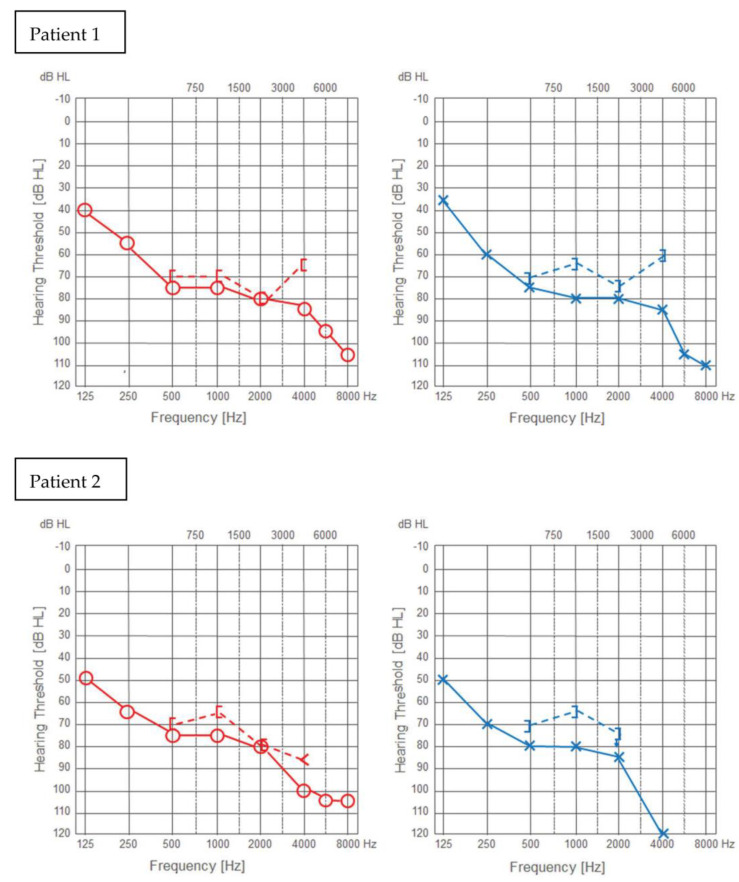
Preoperative audiograms of the patients.

**Figure 3:**
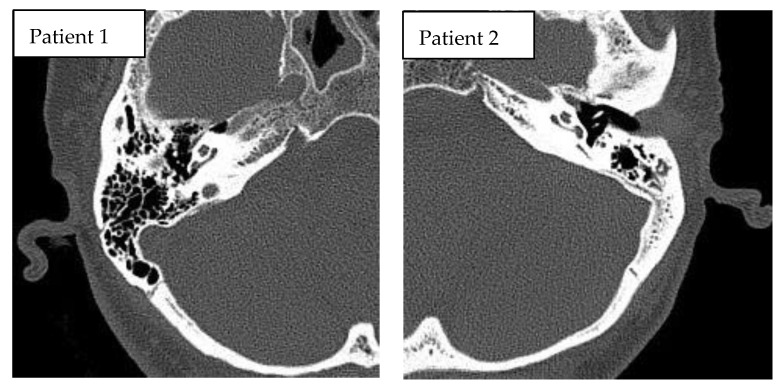
Preoperative CT of temporal bones of the patients, both cases with normal anatomy of the temporal bone.

This prospective, pilot study included two adult patients (females, aged 57 and 61 years) who underwent cochlear implantation in a tertiary referral center. Robot-assisted cochlear implantations were performed on 12–13 July 2022. The surgeries were preceded by surgical training on an artificial temporal bone (Figure 1). The patients had passed the typical procedure for qualifying for a cochlear implant at our center before surgery. Both patients had residual hearing in the implanted ear (Figure 2). The preoperative CT of the temporal bone (Siemens, Somatom Definition Edge, Munich, Germany) showed normal anatomy of the ear qualified for cochlear implantation in the both cases (case 1–right ear, case 2–left ear) (Figure 3). The patients provided informed, written consent for their participation in the study and the publication of its findings. The study was approved by the local Bioethics Committee (decision number 1033/19).

### 2.2. Types of Device and Electrode Arrays

Both patients had chosen the Advanced Bionics cochlear implant and the same type of electrode array was inserted: HiFocus™ Mid-Scala electrode array. This pre-curved electrode array has an active length of 15.5 mm and 16 electrodes.

### 2.3. Robot-Assisted Electrode Array Insertion

The surgeries were performed with the use of RobOtol^®^ (Collin, Bagneux, France). The RobOtol^®^ arm was controlled by the surgeon using a SpaceMouse^®^ (3DConnexion, Waltham, MA, USA). The speed of the robotic arm could be switched between three gears (high speed: 10 mm/s; medium speed: 2 mm/s; low speed: 0.1–1 mm/s). Before the robot-assisted procedure, RobOtol^®^ was sterile covered, moved into the optimal surgical position and then the Boglock sterilized connector (Collin, Bagneux, France; AB Mid-Scala: RBT-0406) was set on the arm. The internal coil of CI was inserted into the subperiosteal pocket and the transducer was positioned in the bone bed and fixed with surgical thread. Then Mid-Scala array was positioned on the insertion tool previously attached to the dedicated connector and the prepared set was coupled to the robot arm by Boglock.

### 2.4. Surgical Technique

The cochlear implantations were performed by the senior otologist (WG). The same standard surgical approach was used in both cases (via mastoidectomy and posterior tympanotomy). The electrode array was inserted through the round window in the first patient and through the cochleostomy in the second (due to the poorly visible round window).

### 2.5. Cochlear Implant System Activation

The cochlear implant system’s initial activation was carried out one month after the CI surgery.

### 2.6. Electrophysiological Measurements

Typical electrophysiological measurements were performed during surgery (impedances of the electrodes and neural response telemetry) and at initial system activation (impedances of the electrodes). Additionally, intraoperative and postoperative monitoring of CM electrical potentials (ECochG) was performed.

### 2.7. ECochG Measurement

**Figure 4:**
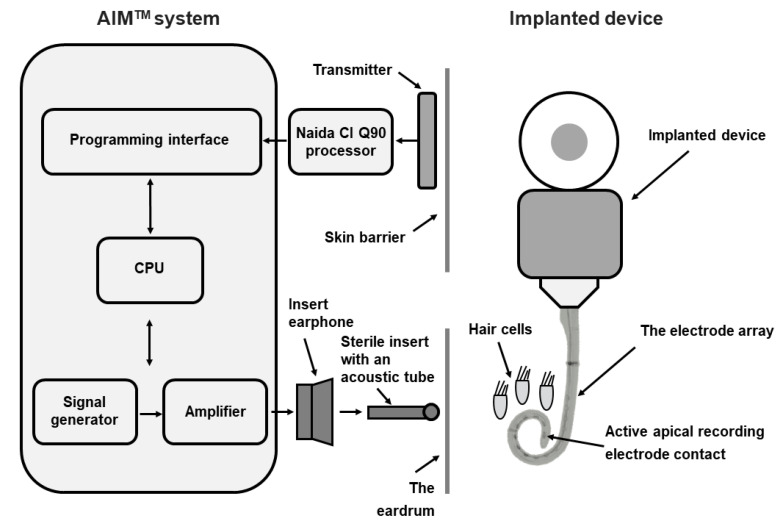
The AIM system schematic block diagram.

Intraoperative and postoperative ECochG measurements were performed with the Advanced Bionics Active Insertion Monitoring AIM^TM^ system. The system consists of the AIM System tablet, Naida CI Q90 sound processor, headpiece, cables, insert earphone and sterile inserts with an acoustic tube. Surgical preparation for the measurement was performed according to the AIM^TM^ system Intra-Operative Guide. During electrode array insertion, a 50 ms tone burst stimulus (500 Hz) of alternating polarity was delivered at 115 dB SPL via the external ear canal with sterile inserts and an acoustic tube. The ECochG responses were recorded with the apical-most electrode contact. The acoustic feedback on changes in ECochG magnitude during the electrode array insertion was automatically provided to the surgeon. The AIM system schematic block system diagram is presented in Figure 4.

After full electrode array insertion, the ECochG responses were recorded with selected active electrode contacts to assess estimated pure-tone thresholds at audiometric frequencies in the 125–4000 Hz range. For this purpose, implemented in the device, an automatic algorithm of the ECochG signal detection and associated gradually decreasing tone burst stimulus were used (subsequently referred to in the text as the estimated audiogram measurement). The same procedure was repeated during the initial system activation.

### 2.8. Imaging

CT of the temporal bone (Siemens, Somatom Definition Edge, Munich, Germany) was performed the day after surgery to confirm the proper position of the electrode.

### 2.9. Pure-Tone Audiometry

The pure-tone audiometry measurements (Interacoustics AC40) were performed preoperatively and postoperatively to assess air conduction (AC) and bone conduction (BC) thresholds in line with ISO 8253–1:2010 standards in selected periods, no earlier than one month before surgery (AC and BC thresholds), two days after the surgery (BC thresholds only), and 1-month after the surgery (AC and BC thresholds).

## 3. Results

### 3.1. Intraoperative Course

**Figure 5:**
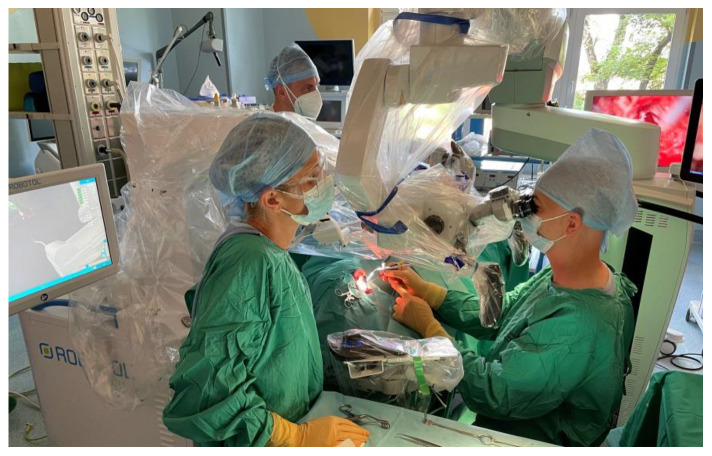
The RobOtol^®^ system is ready to use. The insertion tool is attached to a dedicated connector and coupled to the robot arm by Boglock to confirm the optimal position of the robot arm.

**Figure 6:**
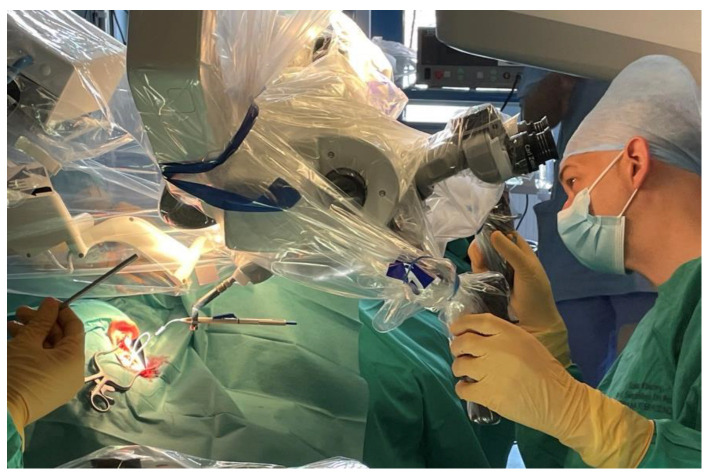
The Mid-Scala array is positioned on the insertion tool and connected to the robot. The system is ready for electrode array insertion.

**Figure 7:**
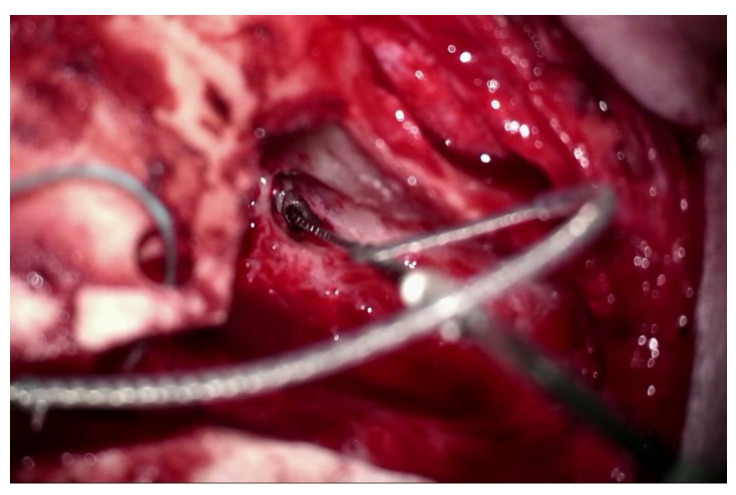
The electrode array insertion via typical (antromastoidectomy and facial recess) approach with a RobOtol^®^ (case 1).

The surgery was uneventful for both patients. The approach to the cochlea was typically completed-by antro-mastoidectomy and posterior tympanotomy. The bone bed for the transducer was drilled. The cochlea was opened by the round window in the first patient and by performing cochleostomy (due to poor visibility of the round window) in the second. The internal coil of CI was inserted into the subperiosteal pocket and the transducer was positioned in the bone bed and fixed with surgical thread. Then the insertion tool was connected to a dedicated connector and coupled for a moment to the robot arm by Boglock (Collin, Bagneux, France; AB Mid-Scala: RBT-0406) to confirm the optimal position of the robot arm (Figure 5), and then decoupled. In the next stage, the Mid-Scala array was positioned on the insertion tool and connected to the robot (Figure 6). The electrode was moved directly to the cochlear opening and slowly inserted to the first blue marker using a robot (Figure 7). Further insertion was carried out by hand with a slider on the insertion tool. However, the stable position of the tool allowed for a very slow and gentle insertion. Moreover, it was possible to stop the electrode insertion and keep it in one position for a few or even several seconds if the ECochG potential decreased. What is more, the insertion axis of the electrode array was slightly modified when iECochG potentials decreased in case one, which improved the iECochG results. The full insertion (till the second blue marker) was carried out in both cases. Then the intraoperative measurements were completed. The connecting cable to the electrode was positioned in the antro-mastoidectomy and the wound was typically closed.

### 3.2. Intraoperative Electrocochleography

**Figure 8:**
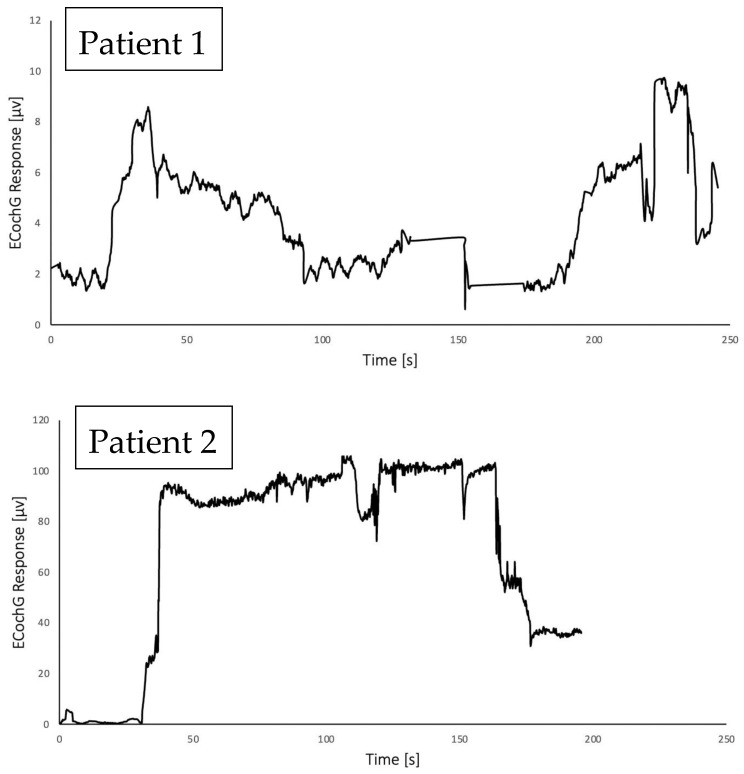
The intraoperative ECochG response measurements for patient 1 and patient 2.

The results of intraoperative ECochG measurements are presented in Figure 8. The ECochG responses reflected the electro-mechanical activity of the inner ear on acoustic stimulation during the insertion of the electrode array into the cochlea. Ideally, the signal amplitude is expected to increase to some extent as the electrode array`s most apical electrode contacts approach the cochlea’s signal source. After bypassing the hair cells, which are probably responsible for the generated signal, the amplitude should gradually decrease with distance from this site. In fact, every movement of the electrode array can cause substantial disturbance in the micromechanical characteristic of the inner ear. Figure 8 shows the ECochG signal waveforms recorded for the two subjects who underwent CI surgery supported with the AIM system measurement. The observed changes in the amplitude of the ECochG signal are presumably results of electrode movement toward the cochlea, unintentional and unpredictable physical contact of the basilar membrane with the electrode array, slight movement of the robotic arm and surgeon’s hand, as well as the implemented measurement technique. The maximum value of the recorded signal may vary for each subject. A credible ECochG response is considered to exceed 3 µV (internal noise of the implantable system does not exceed around 1 µV). The ECochG response was above the mentioned value after complete electrode insertion in both cases.

### 3.3. Imaging

**Figure 9:**
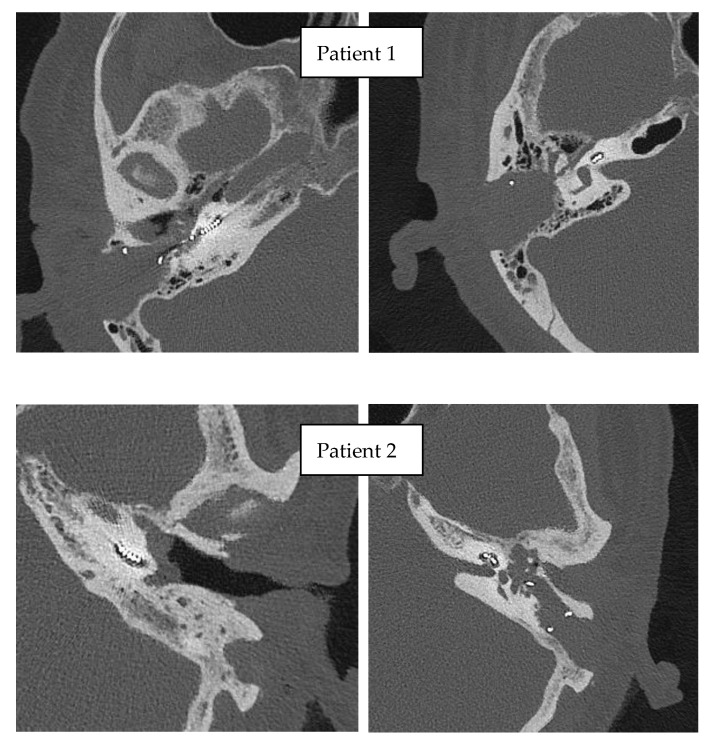
Postoperative CT scans of operated patients.

The post-operative CT confirmed the intracochlear position of the electrode arrays (with the tip of the electrode array in the medial turn of the cochlea) in both patients (Figure 9).

### 3.4. Pure Tone Audiometry and the Estimated Audiogram

**Figure 10:**
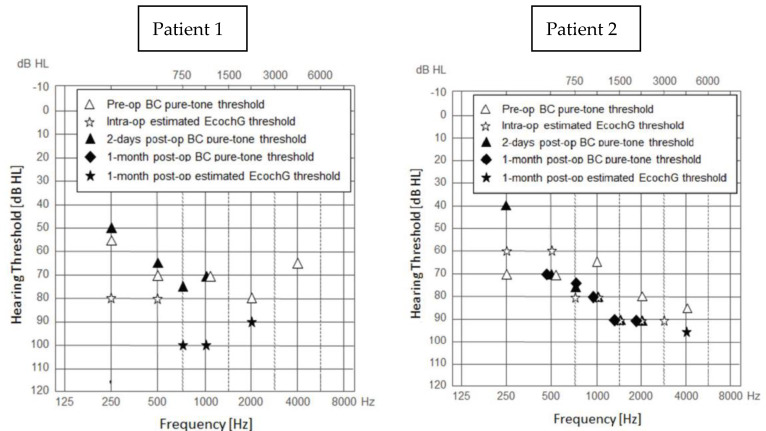
The patients’ preoperative BC thresholds (triangles), intraoperative estimated ECochG thresholds (asterisks), 1-month postoperative estimated ECochG threshold (solid asterisks), postoperative BC thresholds after 2 days (solid triangles) and 1 month (solid diamonds).

The results of pure tone audiometry and the estimated audiograms are presented in Figure 10. Preoperative BC thresholds compared to intraoperative estimated ECochG thresholds and 2-day postoperative BC thresholds had similar values at frequencies where all thresholds were measurable. However, for patient 1 the estimated threshold for 250 Hz seems an outlier (80 dB HL vs. 55 and 50 dB HL). The results of the ECochG performed one month after the surgery showed that in both patients the hearing residues were preserved for the selected frequencies. Moreover, in patient 2, the pure-tone audiometry results confirmed the maintenance of postoperative auditory thresholds for most frequencies.

## 4. Discussion

Cochlear implantation can benefit from robotic assistance in several steps of the surgical procedure: (i) the approach to the middle ear by automated mastoidectomy and posterior tympanotomy or through a tunnel from the postauricular skin to the middle ear (i.e., direct cochlear access); (ii) a minimally invasive cochleostomy by a robot-assisted drilling tool; (iii) alignment of the correct insertion axis on the basal cochlear turn; (iv) insertion of the electrode array with a motorized insertion tool [10]. Currently, there are four systems for clinical robotic cochlear implantation available. Three of them, Microtable^®^ (Vanderbilt), HEARO^®^ (Bern) and ROSA^®^ (Amiens), are used for direct cochlear access but the number of cases implanted with these devices is still very limited [10,23,24,25,26]. The fourth system (RobOtol^®^) is not intended for drilling, but for robotic alignment of the electrode array and its insertion into the scala tympani. This system is clinically used in many European countries, mainly France and in China. More than 250 cochlear implantations with this system have been performed, both in adults and children [10,27,28,29].

The primary assumption of introducing RobOtol^®^ was to optimize the electrode array insertion into the scala tympani and preserve the anatomical structures of the cochlea, which can have many benefits, mainly in patients with residual hearing. It was supposed to have an effect in better hearing if damage to the basilar membrane is avoided and residual hearing preservation is possible [30]. Currently, the RobOtol^®^ can be used with many straight electrodes: SlimJ (Advanced Bionics), 522 and 622 (Cochlear), Flex (Medel), Evo (Oticon) and with one perimodiolar electrode–MidScala (Advanced Bionics) [5,6,9,13,14,28]. The system shows its advantage in eliminating human involuntary tremors and augmenting accuracy during micromanipulation. It can safely assist cochlear implantation to realize minimally invasive and full tympanic scala insertion of the electrode array and to ensure the preservation of the fine intracochlear structure [12]. Despite the promising results in laboratory tests in terms of minimal invasiveness, reduced trauma and better hearing preservation, so far, no clinical benefits on residual hearing preservation or better speech performance have been demonstrated [10]. It is emphasized that new robotic insertion tools should be provided with loop feedback systems capable of modifying the insertion parameters based on both insertion forces and ECochG responses. A preliminary study in vivo sheep model tested the feasibility of an ECochG-guided robotics-assisted CI insertion system [31].

The main goal of our study was to show the application of robotic electrode insertion with simultaneous iECochG measurements, which constituted intraoperative control. We wanted to clarify how the iECochG can improve the robotic cochlear electrode array insertion. To the best of our knowledge, the association between an innovative method of supporting CI surgery and an equally current method of tracking the intraoperative effect has not been published so far. During the electrode insertion with a RobOtol^®^ in our patients with residual hearing, the continuous measurements of ECochG responses were recorded and the insertion speed and axis were constantly modified. For example, electrode insertion was slowed down or interrupted and the insertion axis of the electrode array was modified as the ECochG potential decreased. Perhaps thanks to this, we have managed to partially preserve residual hearing, confirmed by estimated audiograms at the end of the surgeries and during the system’s activation and by measuring bone conduction thresholds by pure tone audiometry. However, our patients require longer follow-ups and subsequent measurements to explain the differences between obtained results and long-term effects.

### Study Limitations

In this work, we wanted to show the possibilities of combining robotic surgery of cochlear implants with electrocochleographic measurement. However, we are aware of the limitations of our work, i.e., a small number of patients and a short observation time.

## 5. Conclusions

The RobOtol^®^ surgical robot allows for the correct, safe and gentle insertion of the cochlear implant electrode inside the cochlea. The use of electrocochleography measurements during robotic cochlear implantation offers an additional opportunity to evaluate and modify the electrode array insertion on an ongoing basis, which may contribute to the preservation of residual hearing.

## Data Availability

The data presented in this study are available on request from the corresponding author.

## References

[1] Bell B., Gerber N., Williamson T., Gavaghan K., Wimmer W., Caversaccio M., Weber S. (2013). In vitro accuracy evaluation of image-guided robot system for direct cochlear access. Otol. Neurotol..

[2] Kratchman L.B., Blachon G.S., Withrow T.J., Balachandran R., Labadie R.F., Webster R.J. (2011). Design of a bone-attached parallel robot for percutaneous cochlear implantation. IEEE Trans. Biomed. Eng..

[3] Riojas K.E., Labadie R.F. (2020). Robotic Ear Surgery. Otolaryngol. Clin. N. Am..

[4] Veleur M., Lahlou G., Torres R., Daoudi H., Mosnier I., Ferrary E., Sterkers O., Nguyen Y. (2021). Robot-Assisted Middle Ear Endoscopic Surgery: Preliminary Results on 37 Patients. Front. Surg..

[5] Vittoria S., Lahlou G., Torres R., Daoudi H., Mosnier I., Mazalaigue S., Ferrary E., Nguyen Y., Sterkers O. (2021). Robot-based assistance in middle ear surgery and cochlear implantation: First clinical report. Eur. Arch. Oto-Rhino-Laryngol..

[6] Daoudi H., Torres R., Mazalaigue S., Sterkers O., Ferrary E., Nguyen Y. (2021). Analysis of forces during robot-assisted and manual manipulations of mobile and fixed footplate in temporal bone specimens. Eur. Arch. Oto-Rhino-Laryngol..

[7] Nguyen Y., Bernardeschi D., Sterkers O. (2018). Potential of Robot-Based Surgery for Otosclerosis Surgery. Otolaryngol. Clin. N. Am.

[8] Kazmitcheff G., Nguyen Y., Miroir M., Péan F., Ferrary E., Cotin S., Sterkers O., Duriez C. (2014). Middle-ear microsurgery simulation to improve new robotic procedures. BioMed Res. Int..

[9] Panara K., Shahal D., Mittal R., Eshraghi A.A. (2021). Robotics for Cochlear Implantation Surgery: Challenges and Opportunities. Otol. Neurotol..

[10] De Seta D., Daoudi H., Torres R., Ferrary E., Sterkers O., Nguyen Y. (2022). Robotics, automation, active electrode arrays, and new devices for cochlear implantation: A contemporary review. Hear. Res..

[11] Loth A., Vazzana C., Leinung M., Guderian D., Issing C., Baumann U., Stöver T. (2022). Quality control in cochlear implant therapy: Clinical practice guidelines and registries in European countries. Eur. Arch. Oto-Rhino-Laryngol..

[12] Jia H., Pan J.X., Li Y., Zhang Z.H., Tan H.Y., Wang Z.Y., Wu H. (2020). Preliminary application of robot-assisted electrode insertion in cochlear implantation. Zhonghua Er Bi Yan Hou Tou Jing Wai Ke Za Zhi Chin. J. Otorhinolaryngol. Head Neck Surg..

[13] Torres R., Daoudi H., Lahlou G., Sterkers O., Ferrary E., Mosnier I., Nguyen Y. (2021). Restoration of High Frequency Auditory Perception After Robot-Assisted or Manual Cochlear Implantation in Profoundly Deaf Adults Improves Speech Recognition. Front. Surg..

[14] Torres R., Hochet B., Daoudi H., Carré F., Mosnier I., Sterkers O., Ferrary E., Nguyen Y. (2022). Atraumatic Insertion of a Cochlear Implant Pre-Curved Electrode Array by a Robot-Automated Alignment with the Coiling Direction of the Scala Tympani. Audiol. Neurootol..

[15] Giardina C.K., Brown K.D., Adunka O.F., Buchman C.A., Hutson K.A., Pillsbury H.C., Fitzpatrick D.C. (2019). Intracochlear Electrocochleography: Response Patterns During Cochlear Implantation and Hearing Preservation. Ear Hear..

[16] Buechner A., Bardt M., Haumann S., Geissler G., Salcher R., Lenarz T. (2022). Clinical experiences with intraoperative electrocochleography in cochlear implant recipients and its potential to reduce insertion trauma and improve postoperative hearing preservation. PLoS ONE.

[17] Koka K., Riggs W.J., Dwyer R., Holder J.T., Noble J.H., Dawant B.M., Ortmann A., Valenzuela C.V., Mattingly J.K., Harris M.M. (2018). Intra-Cochlear Electrocochleography During Cochear Implant Electrode Insertion Is Predictive of Final Scalar Location. Otol. Neurotol..

[18] Pienkowski M., Adunka O.F., Lichtenhan J.T. (2018). Editorial: New Advances in Electrocochleography for Clinical and Basic Investigation. Front. Neurosci..

[19] Riggs W.J., Roche J.P., Giardina C.K., Harris M.S., Bastian Z.J., Fontenot T.E., Buchman C.A., Brown K.D., Adunka O.F., Fitzpatrick D.C. (2017). Intraoperative Electrocochleographic Characteristics of Auditory Neuropathy Spectrum Disorder in Cochlear Implant Subjects. Front. Neurosci..

[20] Harris M.S., Riggs W.J., Giardina C.K., O’Connell B.P., Holder J.T., Dwyer R.T., Koka K., Labadie R.F., Fitzpatrick D.C., Adunka O.F. (2017). Patterns Seen During Electrode Insertion Using Intracochlear Electrocochleography Obtained Directly Through a Cochlear Implant. Otol. Neurotol..

[21] Attias J., Ulanovski D., Hilly O., Greenstein T., Sokolov M., HabibAllah S., Mormer H., Raveh E. (2020). Postoperative Intracochlear Electrocochleography in Pediatric Cochlear Implant Recipients: Association to Audiometric Thresholds and Auditory Performance. Ear Hear..

[22] O’Connell B.P., Holder J.T., Dwyer R.T., Gifford R.H., Noble J.H., Bennett M.L., Rivas A., Wanna G.B., Haynes D.S., Labadie R.F. (2017). Intra- and Postoperative Electrocochleography May Be Predictive of Final Electrode Position and Postoperative Hearing Preservation. Front. Neurosci..

[23] Caversaccio M., Wimmer W., Anso J., Mantokoudis G., Gerber N., Rathgeb C., Schneider D., Hermann J., Wagner F., Scheidegger O. (2019). Robotic middle ear access for cochlear implantation: First in man. PLoS ONE.

[24] Labadie R.F., Balachandran R., Noble J.H., Blachon G.S., Mitchell J.E., Reda F.A., Dawant B.M., Fitzpatrick J.M. (2014). Minimally invasive image-guided cochlear implantation surgery: First report of clinical implementation. Laryngoscope.

[25] Labadie R.F., Riojas K., Von Wahlde K., Mitchell J., Bruns T., Webster R., Dawant B., Fitzpatrick J.M., Noble J. (2021). Clinical Implementation of Second-generation Minimally Invasive Image-guided Cochlear Implantation Surgery. Otol. Neurotol..

[26] Klopp-Dutote N., Lefranc M., Strunski V., Page C. (2021). Minimally invasive fully ROBOT-assisted cochlear implantation in humans: Preliminary results in five consecutive patients. Clin. Otolaryngol..

[27] Daoudi H., Lahlou G., Torres R., Sterkers O., Lefeuvre V., Ferrary E., Mosnier I., Nguyen Y. (2021). Robot-assisted cochlear implant electrode array insertion in adults: A comparative study with manual insertion. Otol. Neurotol..

[28] Barriat S., Peigneux N., Duran U., Camby S., Lefebvre P.P. (2021). The Use of a Robot to Insert an Electrode Array of Cochlear Implants in the Cochlea: A Feasibility Study and Preliminary Results. Audiol. Neurootol..

[29] Jia H., Pan J., Gu W., Tan H., Chen Y., Zhang Z., Jiang M., Li Y., Sterkers O., Wu H. (2021). Robot-Assisted Electrode Array Insertion Becomes Available in Pediatric Cochlear Implant Recipients: First Report and an Intra-Individual Study. Front. Surg..

[30] Gifford R.H., Dorman M.F., Skarzynski H., Lorens A., Polak M., Driscoll C.L.W., Peter Roland P., Buchman C.A. (2013). Cochlear implantation with hearing preservation yields significant benefit for speech recognition in complex listening environments. Ear Hear..

[31] Henslee A.M., Kaufmann C.R., Andrick M.D., Reineke P.T., Tejani V.D., Hansen M.R. (2022). Development and Characterization of an Electrocochleography-Guided Robotics-Assisted Cochlear Implant Array Insertion System. Otolaryngol. Head Neck Surg..

